# Control of metabolic predisposition to cardiovascular complications of chronic kidney disease by effervescent calcium magnesium citrate: a feasibility study

**DOI:** 10.1007/s40620-018-0559-2

**Published:** 2018-11-21

**Authors:** Henry Quiñones, Tamim Hamdi, Khashayar Sakhaee, Andreas Pasch, Orson W. Moe, Charles Y. C. Pak

**Affiliations:** 1 Center for Mineral Metabolism and Clinical Research, University of Texas Southwestern Medical Center, Dallas, TX 75390 USA; 2 Divisions of Nephrology, University of Texas Southwestern Medical Center, Dallas, TX 75390 USA; 3 Mineral Metabolism, University of Texas Southwestern Medical Center, Dallas, TX 75390 USA; 4 Calciscon, Nidau-Biel, Switzerland; 5 Department of Internal Medicine, Department of Physiology, University of Texas Southwestern Medical Center, Dallas, TX 75390 USA

**Keywords:** Calciprotein particles, Chronic kidney disease, Cardiovascular complications, Magnesium, Citrate

## Abstract

**Aims:**

Cardiovascular (CV) complications are common in chronic kidney disease (CKD). Numerous metabolic disturbances including hyperphosphatemia, high circulating calciprotein particles (CPP), hyperparathyroidism, metabolic acidosis, and magnesium deficiency are associated with, and likely pathogenic for CV complications in CKD. The goal of this feasibility study was to determine whether effervescent calcium magnesium citrate (EffCaMgCit) ameliorates the aforementioned pathogenic intermediates.

**Methods:**

Nine patients with Stage 3 and nine patients with Stage 5D CKD underwent a randomized crossover study, where they took EffCaMgCit three times daily for 7 days in one phase, and a conventional phosphorus binder calcium acetate (CaAc) three times daily for 7 days in the other phase. Two-hour postprandial blood samples were obtained on the day before and on the 7th day of treatment.

**Results:**

In Stage 5D CKD, EffCaMgCit significantly increased T50 (half time for conversion of primary to secondary CPP) from baseline by 63% (*P* = 0.013), coincident with statistically non-significant declines in serum phosphorus by 25% and in saturation of octacalcium phosphate by 35%; CaAc did not change T50. In Stage 3 CKD, neither EffCaMgCit nor CaAc altered T50. With EffCaMgCit, a significant increase in plasma citrate was accompanied by statistically non-significant increase in serum Mg and phosphate. CaAc was without effect in any of these parameters in Stage 3 CKD. In both Stages 3 and 5D, both drugs significantly reduced serum parathyroid hormone. Only EffCaMgCit significantly increased serum bicarbonate by 3 mM (*P* = 0.015) in Stage 5D.

**Conclusions:**

In Stage 5D, EffCaMgCit inhibited formation of CPP, suppressed PTH, and conferred magnesium and alkali loads. These effects were unique, since they were not observed with CaAc. In Stage 3 CKD, neither of the regimens have any effect. These metabolic changes suggest that EffCaMgCit might be useful in protecting against cardiovascular complications of CKD by ameliorating pathobiologic intermediates.

## Introduction

CKD is widely prevalent globally [1]. The prevalence of CKD increases progressively with advancing age [2]. Nearly one million persons have end stage renal disease and are treated with hemodialysis (Stage 5D) in the United States [3]. Among patients with CKD, the leading cause of death is cardiovascular complications composed of cardiomyopathy and vascular calcification [4].

There is emerging evidence that cardiovascular complications are likely causally linked to mineral derangements in CKD [5–7]. One such pathophysiologic intermediate is the formation of CPP, which are aggregates of calcium phosphate nanoparticles organized with various proteins with the main component being fetuin, wherein insoluble calcium phosphate is “solubilized” by combining and becoming coated with fetuin [8]. High serum CPP has been reported in patients with CKD [9, 10] and CPP might initiate vascular calcification and cardiomyopathy [11] by induce various cellular responses, including production of reactive oxygen species, mitochondrial dysfunction, cell cycle arrest, and cell death [12]. Serum CPP correlates positively with coronary artery calcification score [9], worsening of kidney function [9], and with all-cause mortality among patients with CKD [13]. To tests CPP’s pathogenic role in cardiovascular complications of CKD, it is necessary devise a countermeasure that would inhibit CPP formation as well as overcome cardiovascular complications.

In addition to CPP formation, CKD-MBD has a wide spectrum of abnormalities that may represent pathophysiologic intermediates of CV disease including hyperphosphatemia [14–16], hyperparathyroidism [17], metabolic acidosis [18], and magnesium deficiency [19] based on epidemiologic association as well as experimental evidence. We hypothesized that EffCaMgCit might be a countermeasure to all the pathophysiologic parameters mentioned above (Table 1**)**. Its provision of optimally soluble and bioavailable magnesium and citrate should deter CPP formation [10, 20], and its magnesium and alkali load should also be cardioprotective [21, 22]. The testing of this hypothesis required the demonstration of not only biochemical improvement but also beneficial effects on cardiovascular function and clinical outcome. Acknowledging that such a goal carries a large commitment of resources with uncertain outcome, we decided to first conduct a feasibility study.

**Table 1:** Therapeutic effects of EffCaMgCitrate on pathophysiologic intermediates

Components of EffCaMgCit	Inhibition of CPP	Phosphate binding	PTH suppression	Magnesium supplement	Alkali supplement
Ca		**√**	**√**		
Mg	**√**	**√**		**√**	
Citrate	**√**				**√**

We report here the results of feasibility study in 9 patients with CKD Stage 3, and 9 patients with CKD Stage 5D, who underwent a randomized crossover trial wherein each received EffCaMgCit or CaAcS for 7 days. The striking findings on serum propensity for CPP formation and other metabolic parameters prompted us to share this preliminary report.

## Materials and methods

### Subjects

Patients were recruited from the nephrology clinic in University of Texas Southwestern Medical Center (UT Southwestern) and Joint Venture DaVita-UT Southwestern dialysis practices in Dallas. All protocols were approved by the Institutional Review Boards at UT Southwestern and DaVita and all patients gave informed consent in their chosen language. 9 patients with Stage 3 CKD and 9 patients with Stage 5D CKD on hemodialysis participated in the study. In Stage 3, 7 were men and 2 women, ranging in age from 50 to 80 years (mean 71). Stage 5D comprised 4 men and 5 women, ranging in age from 37 to 65 years (mean 56). None of the patients had kidney stones, bowel disease, hypercalcemia, hypermagnesemia, or hypophosphatemia (serum *P* < 0.8 mM). In Stage 3 CKD, 4 patients had Type II diabetes and 7 had hypertension, while 4 had Type II diabetes and 8 had hypertension with Stage 5D.

Patients stopped their calcium or magnesium supplements and phosphate binders for 1 week after enrollment and before starting the study, and omitted these medications throughout the study period. They underwent a 2-phase crossover trial lasting 1 week per phase, with 1 week or longer washout between phases. In one phase, they took EffCaMgCit one sachet three times daily with meals for a week. In the other phase, they received CaAc with the same dosing frequency and duration. In each group of subjects (Stage 3 or 5D), the sequence of treatment phases was randomized.

Each sachet of EffCaMgCit, containing 7.5 mmoles Ca^2+^, 5 mmoles Mg^2+^, 20 mEq H^+^, and 15 mmoles citrate, was dissolved in water before ingestion. Each sachet of CaAc, containing 7.5 mmoles Ca^2+^ and 15 mmoles acetate, was suspended in water before ingestion. EffCaMgCit and CaAc were purchased from Sterling Pharmaceutical Services (Dupo, IL) as research drugs. Sachets were identical appearance and identified by different lot numbers.

In patients with Stage 3 CKD, a venous blood sample was obtained at 2 h after ingesting a low calcium meal without EffCaMgCit or CaAc, on the day before each phase started (Day 0). On the 7th day of each phase, a venous blood sample was again taken at 2 h after taking a dose of either EffCaMgCit or CaAc with a similar meal. In patients with Stage 5D CKD, blood sample was obtained from the dialysis vascular access 2 h after ingesting a low calcium meal with or without a test medication.

Serum samples were analyzed for metabolic panel (including calcium, phosphorus, and creatinine) by Quest Diagnostics. UT Southwestern’s Mineral Metabolism Laboratory performed serum magnesium (atomic absorption spectrophotometry), citrate (citrate lyase), intact parathyroid hormone (PTH) (immunoassay, Biomerica), and C-carboxyterminal telepeptide (CTX) (immunoassay, Immunodiagnostic Systems). Frozen serum samples were sent to Calciscon (Nidau-Biel, Switzerland) for T50 analysis [10]. To determine serum supersaturation index of octacalcium phosphate (SI OCP), activity product of OCP was calculated accounting for binding of calcium to albumin and globulin and for soluble complexes of calcium and phosphate, using the JESS computer program [23]. Octacalcium phosphate (Ca_4_H(PO_4_)_3_) is a presumed precursor of calcification.

### Statistical analysis

Mixed-effects linear models were used to compare responses between EffCaMgCit and CaAc treatments. These model included factors for treatment, study day, and interaction between treatment and day; the study participant was modeled as a random effect. Contrasts from these models were used to construct pair-wise comparisons and 95% confidence intervals. Treatment sequence was also assessed in the models and of treatment order had no effect. Skewed variables were analyzed after a logarithmic transformation. All statistical analyses were performed using SAS 9.3 (SAS Institute, Cary, NC). Two-sided P values of < 0.05 were considered statistically significant.

## Results

### Serum magnesium, citrate and T50

**Fig. 1:**
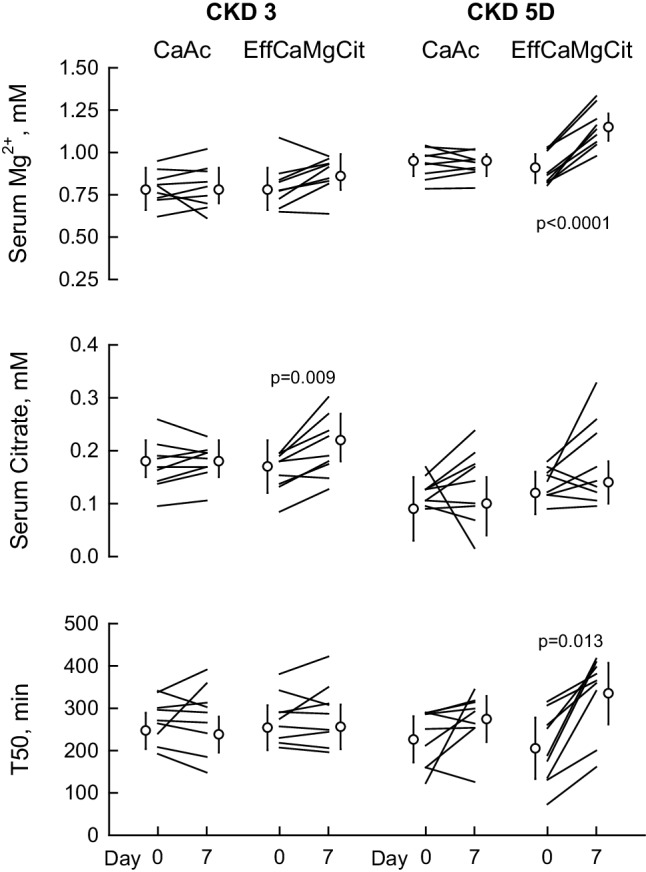
Effect of EffCaMgCit or CaAcS on serum Mg (top), citrate (middle) and T50 (bottom) in CKD Stage 3 (left) and Stage 5D (right). Lines represent paired data from same patients. Symbols and bars indicate the least squares means and 95% confidence intervals. P values for statistically significant differences between Day 0 and Day 7 are shown

In Stage 3 CKD, serum magnesium rose only slightly by 0.08 mM (*P* = 0.07) following EffCaMgCit treatment; it did not change after CaAc (top left, Fig. 1; Table 2). In Stage 5D CKD, however, EffCaMgCit produced a striking increase in serum Mg by 0.24 mM (*P* < 0.0001), whereas CaAc did not (top right, Fig. 1; Table 3). In Stage 3 CKD, EffCaMgCit produced a significant but slight increase in serum citrate whereas CaAc elicited no change (middle left, Fig. 1; Table 2). In Stage 5D CKD, serum citrate was lower than in Stage 3 and it did not change significantly after receiving either EffCaMgCit or CaAc (middle right, Fig. 1; Table 3).

**Table 2:** Laboratory Data in CKD Stage 3

	Calcium Acetate			EffCaMgCit			Interaction
	Day 0	Day 7	P	Day 0	Day 7	P	P
Mg^2+^ (mM)	0.78 (0.66–0.91)	0.78 (0.70–0.91)	0.83	0.78 (0.66–0.91)	0.86 (0.78–0.99)	0.07	0.10
Citrate (mM)	0.18 (0.15–0.22)	0.18 (0.15–0.22)	1.00	0.17 (0.12–0.22)	0.22 (0.18–0.27)	0.009	0.008
T50 (min)	247 (204–289)	238 (195–280)	0.74	254 (201–307)	256 (2–309)	0.96	0.60
P (mM)	1.55 (1.29–1.84)	1.58 (1.29–1.84)	0.89	1.42 (1.19–1.65)	1.61 (1.42–1.84)	0.06	0.06
Ca^2+^ (mM)	2.35 (2.27–2.45)	2.42 (2.35–2.50)	0.10	2.27 (2.17–2.35)	2.42 (2.32–2.52)	0.007	0.06
SI OCP^a^	121 (68–215)	146 (82–259)	0.37	82 (57–118)	148 (103–214)	0.003	0.048
PTH (pM)^a^	9.0 (5.1–16.0)	4.1 (2.3–7.4)	0.003	7.4 (4.1–13.4)	5.0 (2.8–8.9)	0.045	0.07
CTX (ng/L)^a^	460 (190–1090)	130 (50–310)	0.017	390 (250–600)	290 (190–440)	0.17	0.027
CO_2_ (mM)	24 (21–27)	27 (24–29)	0.11	26 (24–27)	25 (23–27)	0.94	0.07
Creatinine (μM)	159 (106–212)	159 (115–212)	0.96	159 (106–212)	168 (115–212)	0.15	0.14

Data represents least squares means or geometric means (95% confidence intervals) from mixed-effects linear models

^a^Geometric means. *Mg*^*2+*^ Magnesium, *T50* measure of propensity for calciprotein particle formation, *P* phosphorus, *Ca*^*2+*^ calcium, *SI OCP* saturation index octacalcium phosphate, *PTH* parathyroid hormone, *CTX* C-telopeptide, *CO*_*2*_ Total carbon dioxide

**Table 3:** Laboratory Data in CKD Stage 5D

	Calcium Acetate			EffCaMgCit			Interaction
	Day 0	Day 7	P	Day 0	Day 7	P	P
Mg^2+^ (mM)	0.95 (0.86–0.99)	0.95 (0.86–0.99)	0.71	0.91 (0.82–0.99)	1.15 (1.07–1.23)	< 0.0001	< 0.0001
Citrate (mM)	0.09 (0.03–0.15)	0.10 (0.04–0.15)	0.62	0.12 (0.08–0.16)	0.14 (0.10–0.18)	0.21	0.54
T50 (min)	226 (172–281)	274 (220–329)	0.14	205 (133–278)	335 (262–407)	0.013	0.010
P (mM)	2.07 (1.61–2.52)	1.55 (1.10–2.00)	0.10	2.32 (1.74–2.87)	1.74 (1.19–2.32)	0.16	0.89
Ca^2+^ (mM)	2.22 (2.12–2.35)	2.37 (2.25–2.47)	0.015	2.22 (2.12–2.32)	2.30 (2.20–2.40)	0.041	0.12
SI OCP^a^	176 (87–355)	117 (58–236)	0.27	211 (114–389)	138 (75–254)	0.20	0.94
PTH (pM)^a^	45.6 (35.3–58.9)	25.7 (19.8–33.2)	0.004	48.7 (38.9–61.0)	29.5 (23.5–36.9)	0.004	0.56
CTX (ng/L)^a^	2410 (1510–3830)	1370 (860–2170)	0.017	2650 (1650–4260)	1780 (1110–2860)	0.07	0.24
CO_2_ (mM)	24 (22–25)	24 (23–26)	0.77	22 (21–24)	25 (24–27)	0.015	0.027
Creatinine (μM)	902 (725–1078)	857 (654–1070)	0.52	902 (725–1078)	893 (681–1096)	0.85	0.44

Data represents least squares means or geometric means (95% confidence intervals) from mixed-effects linear models

^a^Geometric means. *Mg*^*2+*^ magnesium, *T50* measure of propensity for calciprotein particle formation, *P* phosphorus, *Ca*^*2+*^ calcium, *SI OCP* saturation index octacalcium phosphate, *PTH* parathyroid hormone, *CTX* C-telopeptide, *CO*_*2*_ total carbon dioxide

In Stage 3 CKD, T50 was not significantly altered by either EffCaMgCit or CaAc (bottom left, Fig. 1; Table 2). In Stage 5D, however, T50 was markedly increased on EffCaMgCit (63%, *P* = 0.013), but not by CaAc (bottom right, Fig. 1; Table 3).

### Serum phosphate, calcium, and supersaturation index of octacalcium phosphate (SI OCP)

**Fig. 2:**
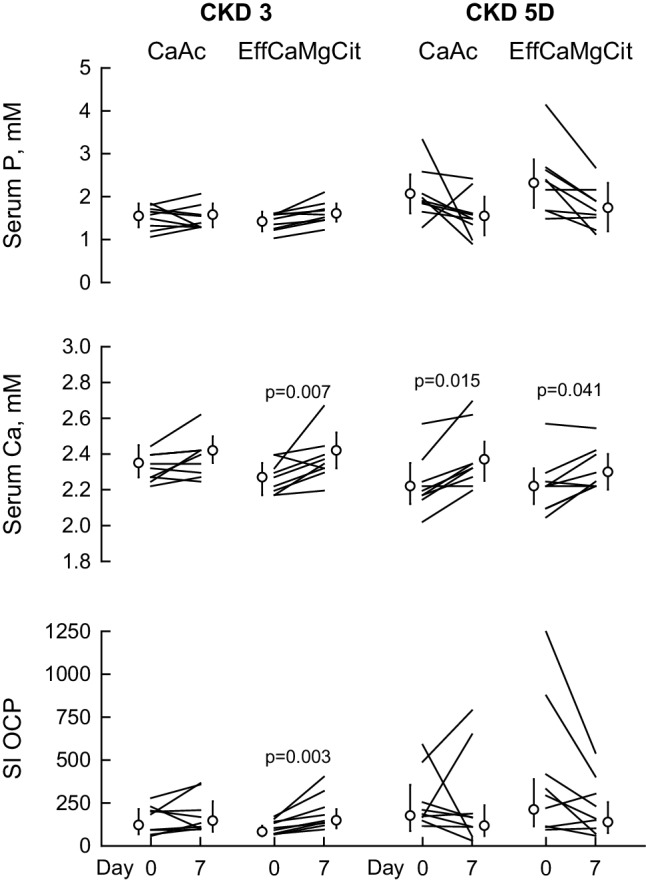
Effect of EffCaMgCit or CaAcS on serum Ca (top), P (middle) and activity product of octacalcium phosphate (SI OCP) (bottom) in CKD Stage 3 (left) and Stage 5d (right). Lines represent paired data from same patient. Symbols and bars indicate the least squares means and 95% confidence intervals. *P* values for statistically significant differences between Day 0 and Day 7 are shown

In Stage 3 CKD, serum phosphate did not change during CaAc but increased numerically but did not reach statistical significance (*P* = 0.06) on EffCaMgCit (top left, Fig. 2; Table 2). In Stage 5D, serum phosphate marginally declined by 0.58 mM from baseline on EffCaMgCit and by 0.52 mM on CaAc (top right, Fig. 2; Table 3).

Both EffCaMgCit and CaAc increased serum Ca in Stage 3 as well as in Stage 5D CKD (middle, Fig. 2; Tables 2, 3). In Stage 3, serum SI OCP did not change on CaAc but rose significantly on EffCaMgCit (bottom left, Fig. 2; Table 1). In Stage 5D, serum SI OCP declined non-significantly on both EffCaMgCit and CaAc (bottom right, Fig. 2; Table 2).

### Serum PTH and bicarbonate

**Fig. 3:**
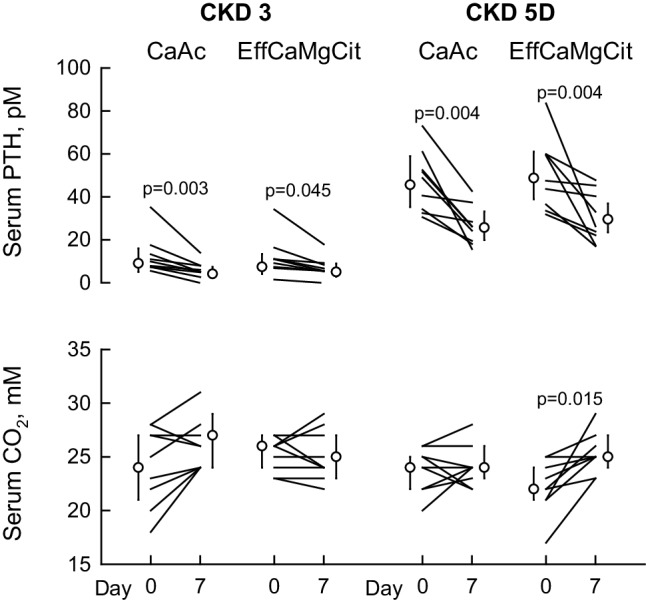
Effect of EffCaMgCit or CaAcS on serum PTH (top) and bicarbonate (bottom) in CKD Stage 3 (left) and Stage 5D (right). Lines represent paired data from same patient. Symbols and bars indicate the least squares means (except for serum PTH, which is geometric means) and 95% confidence intervals. P values for statistically significant differences between Day 0 and Day 7 are shown

In Stage 3 and Stage 5D, both EffCaMgCit and CaAc significantly reduced serum PTH, probably by raising serum Ca (top, Fig. 3; Tables 1, 2). Serum bicarbonate (CO_2_) did not change with either EffCaMgCit or CaAcS in Stage 3 (middle left, Fig. 3; Table 1). However, in Stage 5D, bicarbonate rose significantly on EffCaMgCit but not on CaAc (middle right, Fig. 3; Table 2).

### Other parameters

In Stage 3 CKD, EffCaMgCit marginally reduced serum CTX, and CaAc significantly lowered it (Table 1). In Stage 5D CKD, baseline serum CTX was higher than in Stage 3. Both EffCaMgCit and CaAc significantly reduced serum CTX (Table 2).

In Stage 3 CKD, serum creatinine was modestly elevated at baseline and did not change following treatment with EffCaMgCit or CaAc (Table 1). In Stage 5D CKD, baseline serum creatinine was much higher than in Stage 3. Serum creatinine was unchanged by treatment with either EffCaMgCit or CaAc (Table 2).

## Discussion

EffCaMgCit, a mixture derived from calcium carbonate, magnesium citrate and citric acid, was specially designed to meet the projected goal of this intervention. The amount of Ca^2+^ delivered (7.5 mmoles thrice daily) was sufficient to suppress parathyroid function modestly but not too high to cause adynamic bone disease or increased risk of vascular calcification [24]. The Mg^2+^ content was designed to be just below the threshold of oral Mg^2+^ in the induction of diarrhea [25]. There was citrate excess to H^+^ directed toward conferring a net base load and also to assure complete solubility of Ca^2+^ and Mg^2+^ salts. Calcium acetate was used as a comparator instead of placebo as it would have been unethical not to treat hyperphosphatemia in patients with stage 5D CKD. In stage 3 CKD, use of calcium acetate as a comparator ensured that the effect of the study drug is superior than the standard therapy rather than no therapy. The findings of this feasibility study fulfilled our expectations, with a few surprises.

As expected, EffCaMgCit profoundly increased serum Mg^2+^ in Stage 5D CKD, with a serum Mg^2+^ increment of 0.24 mM (*P* < 0.0001), whereas CaAc did not. This prominent magnesemic response attests to optimum solubility and bioavailability and renal retention of Mg^2+^. In Stage 3 CKD, however, EffCaMgCit elicited only a marginal rise in serum Mg^2+^ of only 0.08 mM (*P* = 0.07). We speculate that the absorbed Mg^2+^ from EffCaMgCit was largely eliminated in urine in subjects with Stage 3 CKD with adequate residual renal function and not in subjects with stage 5D CKD with little or no residual renal function (urinary parameters were not evaluated in these subjects).

In Stage 3 CKD, EffCaMgCit significantly increased serum citrate by 25 mM (*P* < 0.01). In Stage 5D CKD, serum citrate was lower than Stage 3, and increased non-significantly by 0.02 mM following EffCaMgCit treatment. In both Stage 3 and Stage 5D CKD, CaAc did not affect serum citrate.

Our initial hypothesis was that EffCaMgCit might retard CPP formation by increasing both serum Mg^2+^ and citrate thereby inhibiting formation of calcium phosphate (Table 1). In an *in vitro* system, Mg^2+^ delays the conversion of primary CPP (smaller nanoparticles initially formed) to secondary CPP (larger agglomerate) [10]. In the urinary space, citrate is a potent inhibitor of calcium oxalate agglomeration [20]. We believed citrate might have a similar action on calcium phosphate-fetuin agglomeration, and thus CPP formation. The results from the feasibility study suggest that the retained Mg^2+^ seen in the setting of reduced renal function, rather than a change in serum citrate, likely contributed to inhibition of CPP formation. Thus, from the standpoint of inhibition of CPP, CKD Stage 5D appeared to be the ideal target population of EffCaMgCit treatment.

EffCaMgCit might confer cardioprotection in CKD by means other than inhibition of CPP formation. As mentioned above, there are other benefits of EffCaMgCit- namely phosphate binding, Mg^2+^ provision, alkali therapy, and suppression of PTH (Table 1).

EffCaMgCit provides an optimally soluble and bioavailable Mg^2+^ load. In patients undergoing hemodialysis, the well-known serum phosphate-associated increase in cardiovascular mortality was attenuated, in fact nearly abolished, among those in the highest tertile of serum Mg^2+^ concentration [22]. In a meta-analysis of trials on phosphorus binders, the addition of Ca^2+^ appeared to increase, whereas added Mg^2+^ appeared to reduce, the all-cause mortality [24]. In a meta-analysis of various trials, Mg^2+^ supplementation increased serum Mg^2+^ and lowered blood pressure [26]. In patients with CKD, hypomagnesemia was associated with increased mortality and decline in eGFR [27]. Increase in dialysate magnesium level in dialysate decreased serum Calcium precipitation propensity in patients with ESRD [28]. The exact pathogenic mechanism by which Mg^2+^ confers protection against cardiovascular complications in CKD is unknown. It might involve a scheme that is dependent on or independent of inhibition of CPP formation [29].

EffCaMgCit is potentially an efficient phosphate-binding agent, since it delivers already both solubilized calcium and magnesium capable of binding dietary phosphate instantly. In our study, EffCaMgCit reduced serum phosphate by 0.58 mM in Stage 5D; but this change was of marginal statistical significance probably due to low power. CaAc also reduced serum phosphate due to binding by calcium. EffCaMgCit should theoretically be more effective than CaAc due to additional phosphate-binding by Mg^2+^, but this study did not reveal a significant interaction between the two drugs probably due to inadequate power. Owing probably to the decline in serum phosphate (by intestinal binding) and increased complexation of circulating phosphate (by increased serum Mg^2+^), EffCaMgCit reduced the circulating activity product of octacalcium phosphate, a driving force for CPP formation, by 35% but this decline was not statistically significant and was without significant interaction from CaAc. Thus, we conclude that the retardation of CPP formation by EffCaMgCit disclosed in Stage 5d is probably due mostly to the inhibitory effect of increased serum Mg^2+^. It might be partially due to reduced saturation of circulating calcium phosphate, but a larger number of patients would have to be evaluated.

Turning to another potentially beneficial effect of EffCaMgCit, a higher serum bicarbonate associated with a lower incidence of end stage renal disease and small randomized controlled trials have shown retardation of CKD progression [21, 30–35]. EffCaMgCit provide a robust alkali load, since it delivers non-metabolizable cations (calcium and magnesium) and metabolizable anion (citrate). EffCaMgCit increased serum bicarbonate in CKD Stage 5D. EffCaMgCit should be advantageous over the usual alkali therapy since it is devoid of potassium and sodium load which be harmful in CKD.

Lastly, EffCaMgCit used in CKD Stage 5D maintained serum PTH within the recommended desired range of 32 pM, without over-suppression to increase the risk of adynamic bone disease. The amount of calcium delivered by EffCaMgCit in this study (7.5 mmoles per dose three times per day) is lower than from the usual dose of calcium acetate used in CKD [36]. This precaution appeared to produce desired parathyroid suppression without incurring excessive risk of adynamic bone disease.

In conclusion, the strengths of our feasibility trial include demonstrating a potential promise of EffCaMgCit in averting cardiovascular complications of CKD Stage 5D by means of a substantial reduction in the propensity for CPP formation by increasing serum magnesium, with a tendency toward decreasing serum phosphate and saturation with respect to precursor phase of calcium phosphate. Moreover, it provided a potentially beneficial magnesium and alkali load. One of our limitations is the lack of urinary parameters to more objectively explain the lack of these effects in the stage 3 CKD subjects.
